# News and Coming Events

**Published:** 1909-06-19

**Authors:** 


### News and Coming Events.

Professor John Cleland intimated by letter to the Court of the University of Glasgow on June 10 his intention of resigning the Chair of Anatomy, which he has held since 1877.

The Cavendish lecture of the West London Medico-Chirurgical Society will be delivered this year on the subject of "The Cerebellum" by Sir Victor Horsley, F.R.S., F.R.C.S., on Friday, June 25, at 8 p.m., at the Kensington Town Hall. The lecture will be followed by a conversazione.

An extraordinary general meeting of the London and Counties' Medical Protection Society will be held on June 23 at 3.30 p.m. at the offices of the Society, 31 Craven Street, Strand, W.C., for the purpose of considering certain alterations in the articles of association. This will be followed immediately by the annual general meeting of the Society at 4 p.m.

The League of Mercy announces that the Comptroller of H.R.H. the Prince of Wales's Household has issued invitations in the name of T.R.H. the Prince and Princess of Wales, Grand President and Lady Grand President of the League of Mercy, to a reception at Marlborough House on Wednesday, July 7, at four o'clock. If wet the garden reception will not take place.

The conference on the Medical Inspection and Treatment of School Children, under the auspices of the Incorporated Institute of Hygiene, with Sir William Bennett, K.C.V.O., F.R.C.S., in the chair, will commence on Monday June 21, at 4.30 p.m., at 34 Devonshire Street, Harley Street, London, W.

The Medical School of St. Thomas's Hospital will hold its annual prize-giving on Friday, June 25, at 3 p.m. The prizes will be distributed to the students by the Rt. Hon. Alfred Lyttelton, K.C., M.P., in the Governors' Hall, and the ceremony will be followed by tea and music on the terrace.

The Council of the Royal Institute of Public Health have awarded the Harben Gold Medal for eminent services to the public health, which they are empowered to do triennially, to his Excellency Professor E. von Behring, M.D., Marburg, Germany, and have appointed Brevet Lieut.-Colonel W. E. Leishman, M.B., R.A.M.C., Professor of Pathology, Royal Army Medical College, the Harben Lecturer for the year 1910, and Professor Angelo Celli, M.D., Rome, the Harben Lecturer for the year 1911. The Harben Lectures for 1909 will be delivered by Professor R. Pfeiffer, M.D., Breslau, in the Lecture Room of the Institute on Monday, June 21, Wednesday, June 23, and Friday, June 25, at 6 p.m.

The Research Defence Society will hold its annual general meeting of members on Friday, June 25, at the house of the Royal Society of Medicine, 20 Hanover Square, W., at five o'clock, to receive the Committee's report, to confirm the provisional rules, and to transact necessary business. The Earl of Cromer, President, will take the chair and give an address. Other speakers will be Sir James Dewar, Sir Arthur Conan Doyle, the Hon. Walter Guinness, and Professor Starling. The Society has now 2,450 members and 70 associates, and has recently received a legacy of £1,000. The Committee especially wish to make arrangements for more addresses and lantern lectures in various part of the country during the winter. All communications should be addressed to Mr. Stephen Paget, Hon. Secretary, 70 Harley Street, London, W. Telephone: 975 Paddington.

On June 14 Sir Alfred Jones, chairman of the Liverpool School of Tropical Medicine, entertained at dinner in the University Club, Liverpool, twelve members of the school lately returned from scientific expeditions in the tropics. The guests of honour were Professor Ronald Ross, Sir Rubert Boyce, Dr. H. Wolferstan Thomas, Dr. A. Breinl, Professor J. L. Todd, Dr. J. O. W. Barratt, Dr. Warrington Yorke, Dr. A. Kinghorn, Mr. R. E. Montgomery, Mr. R. Newstead, Dr. W. T. Prout, and Dr. A. H. Hanley. The host, in proposing the health of the members of the expeditions, offered on behalf of Liverpool and the Empire generally congratulations and welcome to the members of the various expeditions, who represent not only Great Britain, but other countries, especially Canada. Sir Alfred Jones emphasised the international character of the crusade against tropical diseases, and pointed out with pride that other countries have followed Liverpool's example.

Mr. C. B. Lockwood, of St. Bartholomew's Hospital, occupied the chair at a special meeting of the Motor Union Committee of Medical Motorists held at the offices of the Union, 1 Albemarle Street, Piccadilly, London, W., to consider the Budget proposals of the Chancellor of the Exchequer in so far as they affect members of the medical profession using motor-cars. There was a representative attendance of medical men, members having travelled from the North and West of England in order to be present at the meeting. The committee resolved that the motor-car has now become an absolute necessity to a medical practitioner, who is therefore entitled to make a special claim for abatement. It was resolved to approach the Chancellor of the Exchequer with a view to securing a rebate of 1½d. per gallon on petrol used by medical men. A letter was read from Mr. W. Joynson-Hicks, M.P. (Chairman of the Motor Union), stating that the Chancellor had agreed to receive a deputation on the matter.

### ROYAL COLLEGE OF SURGEONS.

At the last ordinary meeting of the Council of the Royal College of Surgeons of England, held at the College under the chairmanship of Mr. Henry Morris, the President, thirty members of the College, who have recently passed the examinations and conformed to the by-laws, were admitted to the Fellowship of the College; while three candidates, not members of the College, were also admitted Fellows. Following a recommendation from the Committee appointed to consider the subject of instruction in anæsthetics for dental students, the Council determined that all candidates for the licence in dental surgery of the College entering after October 1, 1909, at a recognised dental hospital or school, must produce a certificate "of having attended at a recognised dental hospital and school a course of practical instruction in the administration of such anæsthetics as are in common use in dental surgery."

